# Transcriptome Response Signatures Associated with the Overexpression of a Mitochondrial Uncoupling Protein (AtUCP1) in Tobacco

**DOI:** 10.1371/journal.pone.0130744

**Published:** 2015-06-24

**Authors:** Alessandra Vasconcellos Nunes Laitz, Marcio Luis Acencio, Ilara G. F. Budzinski, Mônica T. V. Labate, Ney Lemke, Paulo Eduardo Martins Ribolla, Ivan G. Maia

**Affiliations:** 1 UNESP, Instituto de Biociências, Departamento de Genética, Botucatu, SP, Brazil; 2 UNESP, Instituto de Biociências, Departamento de Física e Biofísica, Botucatu, SP, Brazil; 3 USP, Departamento de Genética, Escola Superior de Agricultura Luiz de Queiroz, Piracicaba, SP, Brazil; 4 UNESP, Instituto de Biociências, Departamento de Parasitologia, Botucatu, SP, Brazil; Nazarbayev University, KAZAKHSTAN

## Abstract

Mitochondrial inner membrane uncoupling proteins (UCP) dissipate the proton electrochemical gradient established by the respiratory chain, thus affecting the yield of ATP synthesis. UCP overexpression in plants has been correlated with oxidative stress tolerance, improved photosynthetic efficiency and increased mitochondrial biogenesis. This study reports the main transcriptomic responses associated with the overexpression of an UCP (AtUCP1) in tobacco seedlings. Compared to wild-type (WT), AtUCP1 transgenic seedlings showed unaltered ATP levels and higher accumulation of serine. By using RNA-sequencing, a total of 816 differentially expressed genes between the investigated overexpressor lines and the untransformed WT control were identified. Among them, 239 were up-regulated and 577 were down-regulated. As a general response to AtUCP1 overexpression, noticeable changes in the expression of genes involved in energy metabolism and redox homeostasis were detected. A substantial set of differentially expressed genes code for products targeted to the chloroplast and mainly involved in photosynthesis. The overall results demonstrate that the alterations in mitochondrial function provoked by AtUCP1 overexpression require important transcriptomic adjustments to maintain cell homeostasis. Moreover, the occurrence of an important cross-talk between chloroplast and mitochondria, which culminates in the transcriptional regulation of several genes involved in different pathways, was evidenced.

## Introduction

Mitochondria are important sensors of the metabolic and energetic status of the cell. As the center of the respiratory metabolism, mitochondria play a key role in various processes, especially in plant response to stressful conditions [1]. In this scenario, disturbances in the energy status of the cell caused by stress have direct consequences on mitochondrial function, and mitochondrial adjustment to these conditions, culminates in important changes in the cellular activity as well as in nuclear gene expression.

The communication between the mitochondria and nucleus is performed by a mechanism called retrograde signaling, which is already well characterized in yeast, but poorly investigated in plants (reviewed in [2]). Main studies in plants have been performed employing the alternative oxidase (AOX), an important component of the alternative electron transport pathway. It has been demonstrated that the expression of certain genes encoding AOX in *Arabidopsis thaliana* (*AtAOX1a* for example) are induced in response to disturbances in mitochondrial function by retrograde signaling [3,4]. Collected data suggests that AOX is an important marker of mitochondrial dysfunction and cellular oxidative stress, acting in the homeostasis of the signaling molecules involved in organelle to nucleus communication [5]. Moreover, recent results have shown that retrograde communication may also be involved in the regulation of nuclear genes implicated in the biogenesis of the electron transport chain (reviewed in [5]).

The mitochondrial uncoupling protein (UCP) is a component of the mitochondrial alternative pathway in animals and plants (reviewed by [6]). UCPs are located in the inner mitochondrial membrane and catalyze a proton conductance that dissipates the proton electrochemical gradient established by the respiratory chain, thus affecting the yield of ATP synthesis. UCPs are involved in mitochondrial energy flow regulation and reported to play a critical role in the modulation of mitochondrially generated reactive oxygen species (ROS) [6].

Compelling evidences also point to an involvement of UCPs in plant response to stresses, especially those associated with ROS generation [7,8]. In this context, the positive impact of UCP overexpression in plant tolerance to drought and salt stresses has been demonstrated [9]. The observed tolerance was correlated with reduced ROS accumulation in the transgenic plants as a result of increased UCP-mediated mitochondrial uncoupling. However, the exact mechanisms implicated in improved plant performance under stress are still poorly understood. Recently, UCP overexpression has been correlated with increased mitochondrial biogenesis [10].

Due to their involvement in the regulation of the cell redox state and in tolerance to oxidative stress [7,11–13], plant UCPs represent an interesting model for understanding the functional implications of mitochondrial uncoupling in retrograde signaling. In this context, the availability of transgenic tobacco plants overexpressing the UCP1 gene from *A*. *thaliana* (*AtUCP1*) [7], and their respective parental untransformed control, represent an interesting starting point to investigate possible transcriptional changes associated with the constitutive expression of an UCP and to elucidate the mechanisms involved. In a similar approach in mice, it has been observed that the ectopic expression of mammalian UCP3 promotes the up-regulation of genes involved in metabolic response to starvation and fatty acid catabolism in the liver through retrograde signaling [14]. This result, obtained using an animal model, supports the hypothesis that the constitutive expression of an UCP can trigger responses potentially involved in mitochondrial function and retrograde signaling.

In the present study, we established the global changes in gene expression associated with the overexpression of AtUCP1 in entire seedlings of transgenic tobacco using RNA-Sequencing. Interestingly, the transcriptomic responses induced by AtUCP1 overexpression resemble, at least in part, those typically triggered by mitochondrial dysfunction and imply the cross-talk between mitochondria and chloroplast.

## Materials and Methods

### Plant materials and growth conditions

The *Nicotiana tabacum* SR1 plants overexpressing the *AtUCP1* gene used in the present work have been described previously [7]. In this study, two independent and homozygous transgenic lines, termed P32 and P07, which present different levels of *AtUCP1* expression [7] were chosen. Both overexpressors lines (OE) were used as replicas and parental untransformed plants were used as control. The seeds from the transgenic and wild-type (WT) tobacco were surface-sterilized and sown in Petri dishes containing solid Murashige-Skoog (MS) medium. Dishes were maintained in a growth chamber at 20–22°C with a 16/8 h light/dark photoperiod until seedling sampling. For the analyses of RNA-Seq, metabolic profiling, ATP measurement and RT-qPCR, entire 3-week-old seedlings from WT and OE lines (20 seedlings per genotype), respectively, were sampled at the midpoint of the illuminated period.

### ATP measurement

ATP content was determined using the ATP assay kit (Invitrogen) essentially as described [10], with minor modifications. Entire 3-week-old seedlings from WT and OE lines were ground in liquid nitrogen, and 200 mg of tissue powder homogenized in a 4:2:1 methanol/chloroform/water mixture (1 ml). Samples were vortexed, kept on ice for 30 min and vigorously homogenized for additional 15 min. After the addition of water (1 ml), the samples were centrifuged at 12.200 x g for 5 min for phase separation and subsequently dried in a centrifugal vacuum concentrator. The pellet was resuspended in 10 mM phosphate buffer (pH 7.4). Measurement of ATP content was carried out on a GloMax-Multi Detection System (Promega). The obtained data were subjected to ANOVA (t test), and considered significantly different at the P < 0.05 probability level.

### RNA isolation, quality control and sequencing

The sampled seedlings (n = 20) were pooled for total RNA extraction using Trizol reagent (Invitrogen). The RNA samples were then quantified spectrophotometrically using NanoDrop (Thermo Scientific). Total RNA integrity (1 μg) was further evaluated using the RNA 6000 Nano LabChip Kit and a Bioanalyzer 2100 (Agilent Technologies). RNA samples with RNA integrity number ≥ 7 were used for library construction. The sequencing libraries were prepared using the TruSeq RNA Sample Preparation Kit v2 (Illumina). Sequencing was performed on the Illumina MiSeq platform using a paired-end 300 base pair (bp) run. The complete dataset of RNA-Seq reads was deposited in the Sequence Read Archive (SRA) database under accession numbers SRS813418, SRX835497, SRR1747973 and SRR1747974.

### *De novo* transcriptome assembly and mapping

The raw data generated for each sample were separately trimmed and *de novo* assembled in a unique file using the CLC Genomics Workbench software (Version 6.5, CLC Bio). The reads were trimmed using the quality score limit of 0.08 and maximum limit of 2 ambiguous nucleotides, and then assembled using the "de novo assembly" algorithm. The trimmed reads were mapped to the *de novo* assembled contigs with parameters allowing mapping of reads to the genome with up to 3 mismatches. The reads mapped to ribosomal RNA as well as those not uniquely mapped were removed from subsequent analyses. The expression levels were evaluated using the RPKM (reads per kilobase per million) method essentially as previously described [15]. Data accuracy was checked by the calculation of a correlation coefficient using the SigmaStat package based on the log-transformed RPKM values derived from the OE lines dataset and after eliminating genes with zero count.

### Functional annotation of transcripts

Functional annotation was performed by the assignment of Gene Ontology (GO) terms [16] to the transcripts. For this purpose, the Blast2GO suite [17] with default parameters was used. Blast2GO assigns GO terms to sequences through a 3-step process: blasting, mapping and annotation. In the blasting step, sequences are blasted using BLASTX (1e^−5^) against the sequence database provided by the National Center for Biotechnology Information (NCBI). In the mapping step, GO terms are mapped on the blast results using annotation files provided by the GO Consortium. In the annotation step, the GO terms mapped to the blast results are transferred to the target sequences using the default annotation parameters [17]. Differentially expressed genes were also annotated against the Kyoto Encyclopedia of Genes and Genomes (KEGG) [18] using Blast2GO.

### Differential gene expression analysis

The differential gene expression between OE lines and WT was determined using the CLC Genomics Workbench v6.5 (CLC Bio). For this, RPKM values were submitted to quantile normalization and then log_2_-transformed for subsequent statistical analysis. Hierarchical clustering of samples and principal component analysis were carried out to examine data quality and comparability. Aiming the identification of genes with significant changes in expression between the OE lines and WT (P07 vs WT and P32 vs WT), a Kal's *Z-test* was performed on log_2_-transformed data [19]. For that, a 2-fold change cutoff plus a p-value cutoff of 0.001 was employed.

### GO-based functional enrichment analysis

Enrichment analysis was carried out for the differentially expressed transcripts. For each type of GO categories, that is, biological process, molecular function and cellular component, we compared the frequencies of GO terms mapped to the differentially expressed transcripts with those of entire set of expressed transcripts. To this end, The Biological Networks Gene Ontology tool (BiNGO) [20], an open-source Java tool to determine which GO terms are significantly overrepresented in a set of genes, was employed. The statistical test (hypergeometric test), the multiple testing corrections (Benjamini and Hochberg correction) and the confidence level (p < 0.05) were default values. The reference set, the ontology and organism/annotation files were all prepared in a customized way. For each type of ontology, the test set consisted in the set of differentially expressed transcripts and the reference set consisted in the complete set of expressed transcripts.

### Differentially expressed metabolic pathways

To determine which metabolic pathways were differentially expressed between control and the investigated OE lines, we first identified the transcripts likely coding for enzymes using Blast2GO. Besides assigning GO terms, this software also assigns Enzyme Commission numbers (EC numbers) to sequences. The retrieved EC numbers were then mapped to the plant metabolic pathways collected from Gramene [21], an integrated data resource for comparative functional genomics in crops and model plant species. In subsequent analysis, only pathways with at least five mapped EC numbers were kept. Next we mapped back the EC numbers to their coding genes and then calculated the fold change in gene expression in OE lines compared with WT control plants. The fold change is given by log_2_(ExpA/ExpB), where ExpA and ExpB are, respectively, expression values for genes in transgenic and control plants. For each pathway containing *k* enzymes, the average fold change was calculated and statistically compared with the average fold change obtained by 100 sets of *k* enzymes randomly taken from the pathways. From this average fold change and its corresponding standard deviation, a *Z-score* and a *p-value* were calculated for each metabolic pathway. We considered differentially expressed the pathways with p-value <0.05. Positive and negative *Z-scores* indicate, respectively, up-regulated and down-regulated pathways.

### Validation of the RNA-Seq results by RT-qPCR

In order to verify the accuracy of the expression profiles determined by RNA-Seq, 17 transcripts were selected for quantitative RT-PCR (RT-qPCR) assay (S1 Table). The RNA (2 μg) of each sample was treated with DNaseI (Fermentas) and the High Capacity cDNA Reverse Transcription Kit (Applied Biosystems) was used for cDNA synthesis following manufacturer’s instructions. All cDNA samples were quantified on a NanoDrop ND-1000 spectrophotometer (NanoDrop Technologies).

Relative gene expression levels were determined by quantitative real-time PCR (qPCR) using a Step One Plus Real-Time PCR System (Applied Biosystems) under the following parameters: 95°C for 10 min, 40 cycles at 94°C for 15 s, 60°C for 60 s. Amplicon specificity was checked using the dissociation curve at the end of each run. Each PCR reaction (12 μl) consisted of 6μl of Maxima SYBR Green/ROX qPCR Master Mix (Thermo Scientific), 1 μl of cDNA and 0.4 μM of forward and reserve primers. All gene-specific primer pairs (S1 Table) were designed using the Primer Express software (Applied Biosystems). In all reactions, the gene encoding an elongation factor (NtEF) from *N*. *tabacum* was used as an internal control to normalize levels of target transcripts (S1 Table). The generated relative expression data were further analyzed using the DataAssist software v.3.01 (Applied Biosystems). Additionally, the correlation between the RNA-Seq and RT-qPCR expression values obtained for the selected genes (total of 17) was evaluated by calculating the Pearson correlation coefficient.

### Metabolite profiling and data analysis

Metabolites were extracted from entire 3-week-old seedlings from WT and OE lines as described [22]. Stable isotope reference compounds [1 mg/ml each of (13C3)-myristic acid and (2H4)-succinic acid] were used as external standard. After extraction, 100 μL of each supernatant were transferred to a GC-vial and evaporated to dryness. The samples were derivatized with 30 μL of methoxyamine hydrochloride (15 mg/ml) in pyridine for 16 h at room temperature (RT). Trimethylsilation was performed by adding 30 μL of N-methyl-N-trimethylsilyltrifluoroacetamide (MSTFA) with 1% TMCS to the samples and incubating them for 1 h at RT. After silylation, 30 μL of heptane was added. Samples were analyzed using gas chromatography with time-of-flight mass spectrometry (GC/TOFMS) with a series of n-alkanes (C12–C40), which allowed retention indices to be calculated [23]. One microlitre of each derivatized sample was injected splitless into a gas chromatograph 7890A (Agilent Technologies) coupled with a Comb-xt Autosampler (Leap Technologies). The injector temperature was 280°C, the septum purge flow rate was 20 ml/min and the purge was turned on after 60 s. The gas flow rate through the column was 1 ml/min, the column temperature was held at 80°C for 2 min, then increased to 305°C (15°C/min), and was held there for 10 min. The column effluent was introduced into the ion source of a GC×GC/TOFMS (Pegasus 4D, Leco, St. Joseph). The transfer line and the ion source temperatures were 280 and 250°C, respectively. Ions were generated by a 70 eV electron beam at an ionization current of 2.0 mA, and 10 spectra s-1 were recorded in the mass range 45–800 m/z. Chromatograms were exported from Leco ChromaTOF software (version 4.51). Peak detection, retention time alignment and library matching were performed using the TargetSearch software package [24]. Metabolites were selected by comparing their retention indexes (±—2 s) and spectra (similarity > 600) against the compounds stored in the Golm-Metabolome-Database (GMD) [25].

Statistical analyses were performed in the Metaboanalyst web portal (www.metaboanalyst.ca) [26]. Metabolite data were normalized by median centering, log_10_-transformed and re-scaled according to Pareto scaling method. The significant differences between WT and transgenic lines were determined by using a Volcano plot (p<0,05).

## Results

In other to get further insights into the broad transcriptional responses associated with the overexpression of an uncoupling protein, global gene expression profiles from 3-week-old seedlings of WT and two AtUCP1 OE lines (P07 and P32) were determined using RNA-Sequencing. At this developmental stage, the OE lines displayed no phenotypic differences when compared to WT plants with the exception of a faster vegetative growth [9]. Most importantly, however, is the fact that at this stage the AtUCP1 OE lines proved to be highly tolerant to salt and drought stresses [9]. It should be also highlighted that the two selected OE lines show different expression levels of the transgene, with P07 being considered a high expression line and P32 a moderate one [7,10].

**Fig 1:**
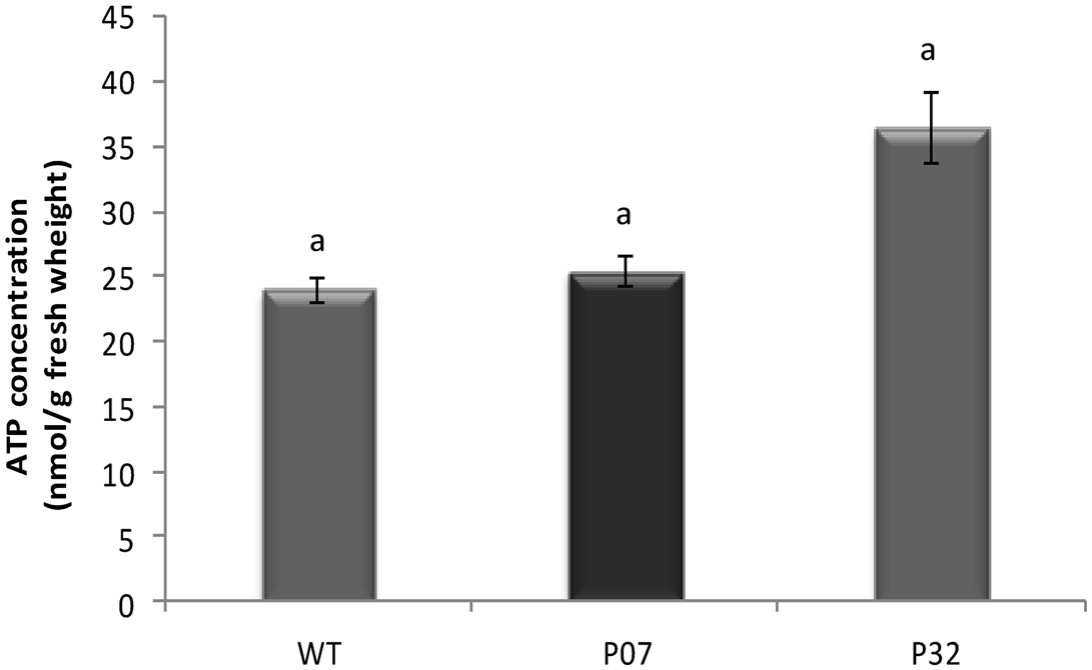
ATP content in intact seedlings of wild-type (WT) and AtUCP1 OE lines (3-week-old). The data are representative of nine replicates. Means with the same letter are not significantly different (t test; p<0.05).

To determine whether UCP overexpression resulted in decreased levels of cellular ATP as previously described in leaves of the P07 OE line [10], we evaluated ATP content in entire 3-week-old seedlings. As shown in Fig 1, no significant differences in the ATP content between WT and AtUCP1 OE lines were observed, indicating that, at the investigated developmental stage, AtUCP1-mediated uncoupling has no negative impact on ATP production.

### Global analysis and functional annotation

De novo assembling was performed by pooling the resulting RNA-Seq data of the investigated samples. The resulting assembling parameters are presented in Table 1. A total of 6.333.375 high quality pair-ended sequencing reads were generated from a 300 bp insert library. Based on the high quality reads, a total of 33.679 contigs were assembled with an average length of 602 bp. The contigs lengths ranged from 197 to 5.386 bp. From the total reads, 68.1% matched with the contigs, and 84.9% of them were found in pairs. Average distance of read-pairs was approximately 145 bp, ranging from 60 to 230 bp.

**Table 1:** Assembling parameters.

Reads	9,777,662
Reads in pairs	6,333,375
Number of contigs (≥200)	33,679
N50 (bp)	632
Maximum Contig length (bp)	5,386
Minimum Contig length (bp)	197
Average (bp)	602

N50 = statistically weighted average such that 50% of the entire assembly is formed by contigs of equal size or larger than this value.

**Fig 2:**
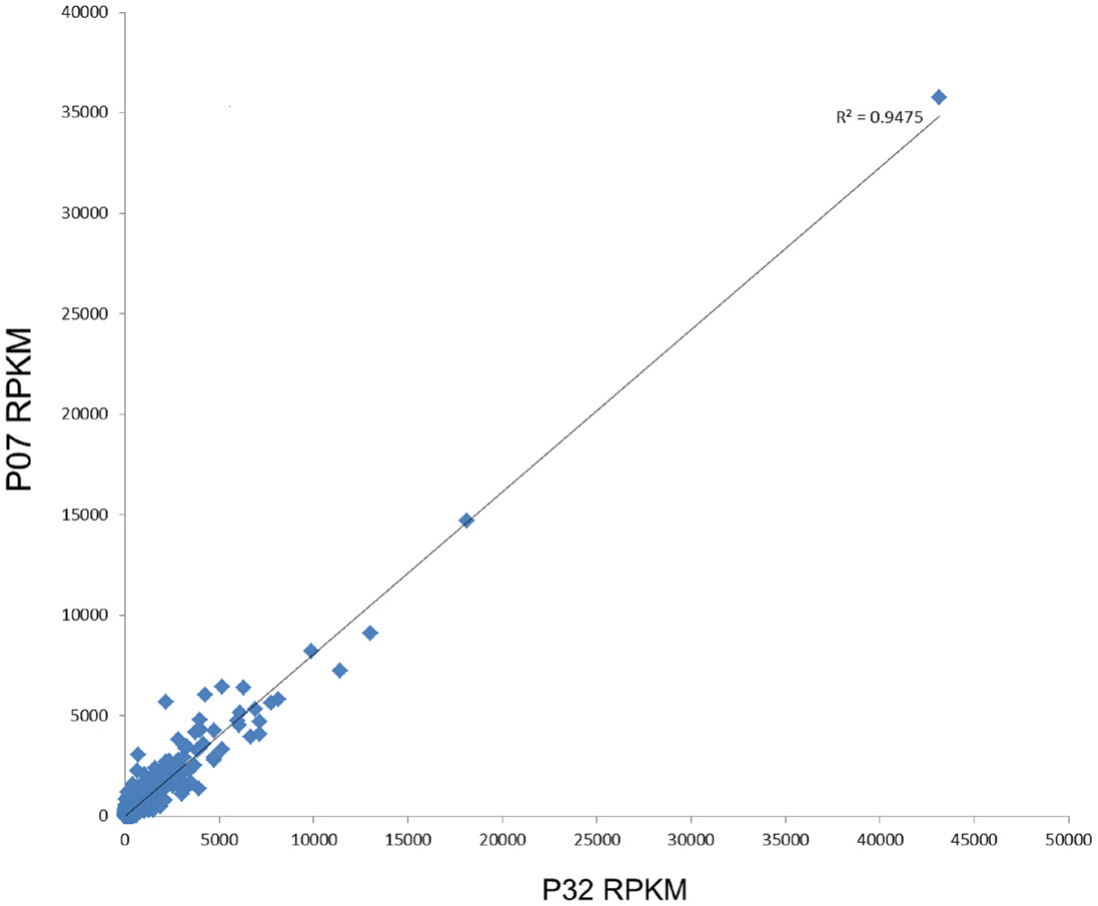
R^2^ linear regression of the RNA-Seq data from both OE lines (P07 and P32). The R^2^ values were calculated using the Sigma Stat package based on the RPKM values derived from RNA-Seq data and after eliminating genes with zero count.

Among the 33.679 assembled contigs, 29.152 (86%) showed positive matches in BLAST searches against the NCBI database and 25.246 (75%) were associated with at least one gene ontology (GO) term when classified using Blast2GO. A relative number of contigs showing different expression levels received the same functional annotation, which likely reflects the presence of closely related paralogs or alternative spliced forms. Moreover, a high correlation (R^2^ = 0.947 with p-value<0.001) between the two OE lines was observed (Fig 2), thus supporting the reliability of the raw dataset. This dataset was considered satisfactory to investigate the main transcriptomic effects of AtUCP1 overexpression in transgenic tobacco.

### Differential gene expression and GO enrichment analysis

By using a 2-fold change cutoff plus a p-value cutoff of 0.001, a total of 816 differentially expressed genes (DEGs) between the AtUCP1 OE lines and WT plants was detected, among which 239 were up-regulated and 577 were down-regulated (full list available in S2 Table). It should be emphasized that only DEGs that were in common between both OE lines were considered. As expected, *AtUCP1* was the most up-regulated gene in both transgenic lines by more than 700-fold as compared to WT (S2 Table).

**Fig 3:**
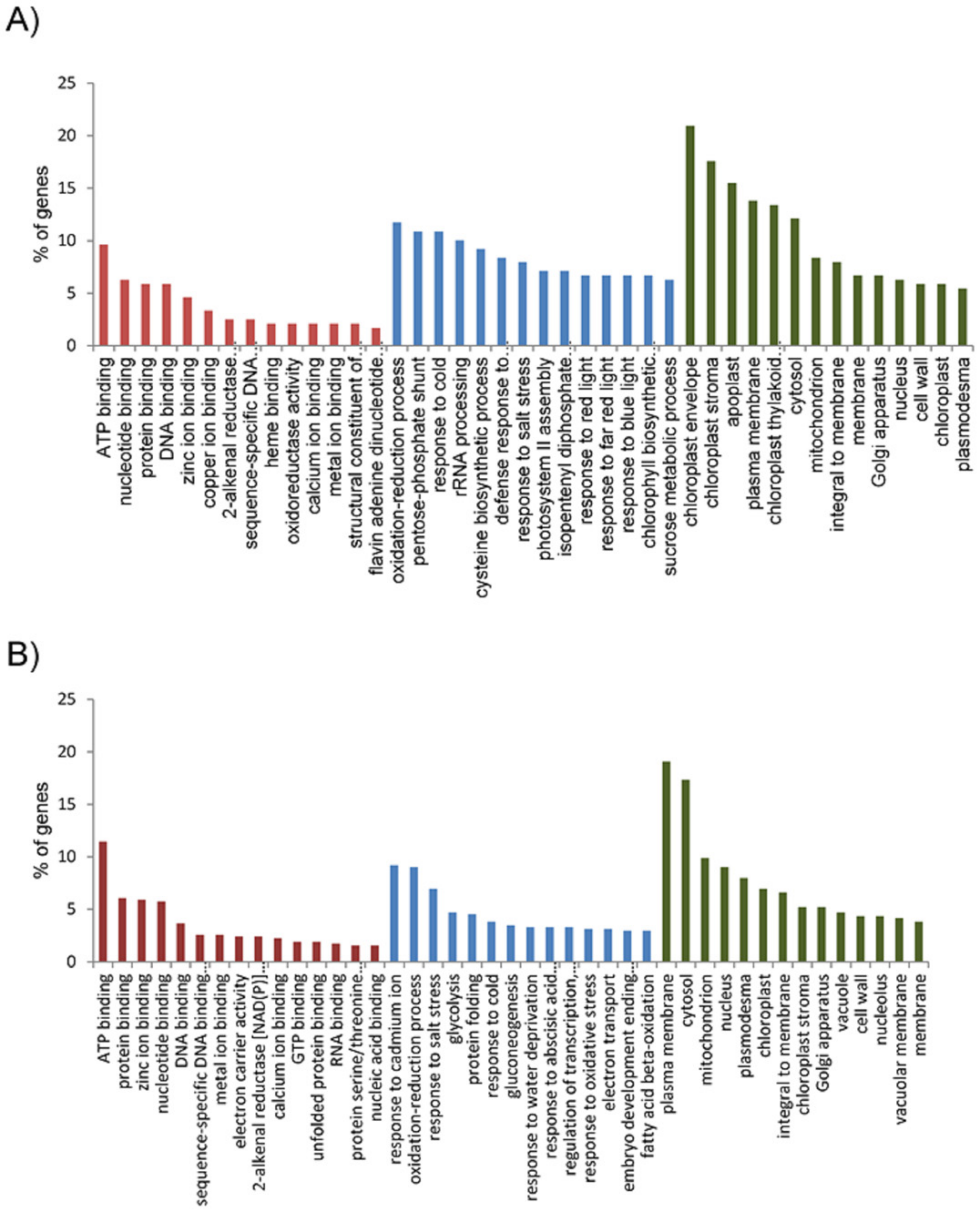
Gene Ontology (GO) analysis of the differentially expressed genes. Distribution histograms within the three main GO categories (in red “molecular function”, in blue “biological process” and in green “cellular component”) are shown for the up- (A) and down-regulated (B) genes. Only differentially expressed genes that were in common between the two AtUCP1 OE lines (P07 and P32) were included in the analysis.

As a starting point to functional annotation, we first determined the GO terms assigned to both down- and up-regulated genes (Fig 3). It should be mentioned that in many cases, different GO terms were assigned to the same transcript. For the up-regulated genes (Fig 3A), “ATP binding” (23 transcripts—9.62%) and “nucleotide binding” (15 transcripts—6.27%) were the most predominant GO terms in the “molecular function” category, while “oxidation-reduction process” (28 transcripts—11.71%) and “pentose-phosphate shunt” (25 transcripts—10.87%) were prominent in the “biological process” category. In the “cellular compartment” category, the terms “chloroplast envelope” (50 transcripts—20.92%) and “chloroplast stroma” (42 transcripts—17.57%) were overrepresented.

For the down-regulated genes (Fig 3B), the terms “ATP binding” (66 transcripts—11.43%), “response to cadmium ion” (53 transcripts—9.18%), and “plasma membrane” (110 transcripts—19.06%) were prominent in each of the three main functional categories, respectively. It is also noteworthy the presence of a large number of down-regulated genes associated with the terms “oxidation-reduction process” (52 transcripts—9%) in the “biological process” category, and “cytosol” (110 transcripts—17.33%) followed by “mitochondrion” (57 transcripts—9.87%) in the “cellular compartment” category.

A GO enrichment analysis was subsequently employed to indentify the most representative ontologies associated to each DEG dataset. By using a Hypergeometric test with Benjamini and Hochberg correction through BiNGO [20], we compared, for each dataset, the frequencies of GO terms mapped to the up- and down-regulated genes with those of entire set of expressed genes. By doing so, we determined the GO terms significantly enriched (FDR-corrected for p<0.05) in the up-regulated and down-regulated datasets as compared to all expressed genes.

**Fig 4:**
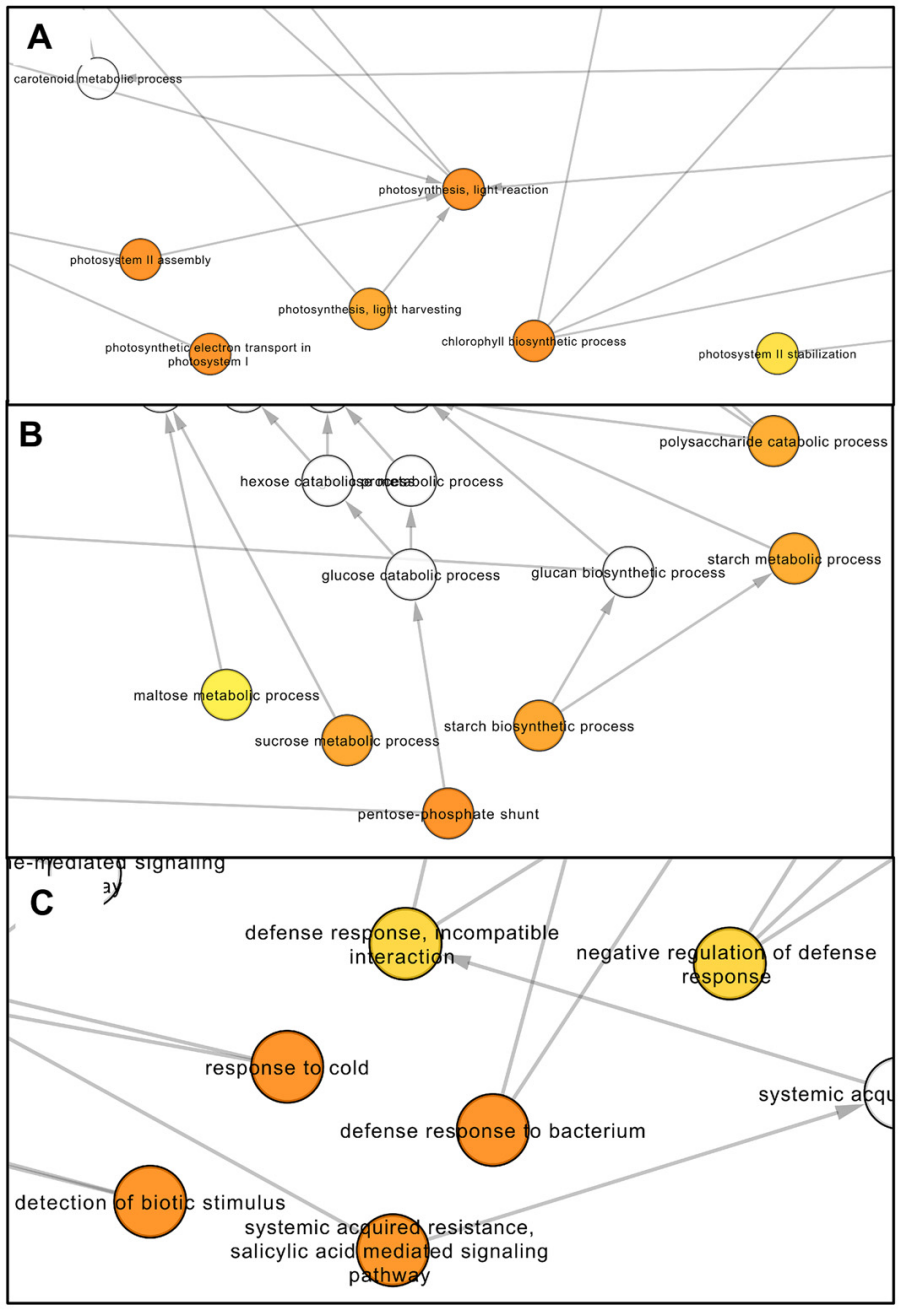
Enriched network based on GO terms in the up-regulated gene dataset associated with photosynthesis (A), carbon metabolism (B) and biotic and cold stress responses (C). Data were analyzed using the Biological Networks Gene Ontology tool (BiNGO). The area of a node is proportional to the number of up-regulated genes annotated to the corresponding GO category. Node color means enrichment significance *i*.*e*. darker the color on a color scale ranging from white (no significant) to orange (FDR-corrected p-value = 3 x 10^−10^), higher is the enrichment significance. White color nodes are not enriched but show the hierarchical relationship among the enriched ontology branches.

For the up-regulated dataset, the enrichment analysis indicated a total of 69 and 12 significantly enriched GO terms associated with “biological process” and “cellular component” categories, respectively (S3 Table). A graphical illustration of the results of the GO enrichment analysis for the “biological process” category is available in S1 Fig. In this category, the terms “pentose-phosphate shunt” and “rRNA processing” were the most significantly enriched followed by “cysteine biosynthetic process”. Notably, several terms associated with photosynthesis (photosynthesis, light reaction; photosystem II assembly; photosystem II stabilization; photosynthetic electron transport in photosystem I) were overrepresented in the up-regulated gene dataset (detailed view in Fig 4A). This dataset was also significantly enriched for genes linked to chlorophyll (tetrapyrrole) biosynthesis and carbohydrate metabolism (Fig 4B).

Consonant with the observed enrichment of photosynthesis related terms, the majority of the GO terms significantly enriched in the “cellular component” category (S3 Table) was associated with the chloroplast (chloroplast envelope; chloroplast stroma; chloroplast thylakoid membrane; chloroplast thylakoid lumen). Overall, these results indicate an important effect of AtUCP1 overexpression on chloroplast metabolism and confirm the previously reported relationship between UCP activity and photosynthetic efficiency [13,27,28]. In this respect, it should be highlighted that the net photosynthetic rates of the AtUCP1 OE lines were higher than WT plants under control conditions [9].

Interestingly, we also observed the enrichment of several GO terms associate with cold and biotic stress responses (defense response to bacteria, systemic acquired resistance, and detection of biotic stimulus) within the set of up-regulated genes (S3 Table; Fig 4C). These results suggest a connection between UCP-mediated uncoupling and the triggering of specific plant stress responses. In fact, changes in transcript abundance of defense related genes in response to perturbations in the electron transport chain have already been observed [29]. In addition, the observed up-regulation of genes related to cold response is in accordance with previous studies reporting a link between the cold induction of nuclear gene expression and decreased efficiency of oxidative phosphorylation [30,31]. It is worthy of note in this context that UCP action can lead to a fast electron flux through the respiratory chain. Last, concerning the “molecular function” category, the only enriched term was “delta12-fatty acid dehydrogenase activity”, which is required for the generation of unusual fatty acids.

For the down-regulated dataset, a total of 13 and 5 terms were significantly enriched in the “biological process” and “cellular component” categories, respectively (S4 Table). Among them, “chlorophyll catabolic process” and “response to cadmium ion” were the most significantly enriched terms followed by “protein folding”. Although unexpected, the overrepresentation of transcripts associated with cadmium response is in line with a proposed role for the alternative respiratory pathway in modulating cadmium-induced stress responses [32]. Considering the terms significantly enriched in the “cellular component” category, the majority was related to “cytosol” followed by “plasmodesma” and “plasma membrane” (S4 Table). Interestingly, GO terms linked to certain abiotic stress responses (response to oxidative stress, response to water deprivation, hyperosmotic responses) and also to fatty acid oxidation were significantly enriched in the down-regulated gene dataset. It seems thus that fatty acid oxidation is lowered in the OE lines in order to compensate for the metabolic alterations promoted by AtUCP1 overexpression. It should be emphasized that fatty acids are important regulators of UCP activity [6].

### Genes involved in oxidative phosphorylation

Due to the described effect of the dissipating activity of overexpressed AtUCP1 on the yield of ATP synthesis [10], we focused attention on the genes encoding products involved in oxidative phosphorylation. Curiously, none of the examined genes figured as up-regulated in our dataset (S2 Table). On the contrary, many genes encoding components of the mitochondrial respiratory chain, such the beta subunit of F_1_F_0_ ATP synthase, the iron-sulfur subunit 2 of succinate dehydrogenase and different subunits of the NADH dehydrogenase complex (flavoprotein 1, iron-sulfur protein 6, 1 alpha subcomplex subunit 13), respectively, were down-regulated in the OE lines (S2 Table). These results, while consistent with the undetected impact of AtUCP1 overexpression on the ATP content of the investigated transgenic seedlings (Fig 1), contrasted those reported previously showing a transcriptional upregulation of the main components of the electron transport chain in the leaves of the P07 OE line [10].

### Altered metabolic pathways in the OE lines

The KEGG database was employed to identify the main metabolic pathways altered in the AtUCP1 OE lines. As a result, 52 KEGG pathways (showing 5 or more altered genes) were affected by AtUCP1 overexpression (S5 Table). Amongst them, “starch and sucrose metabolism” was the most affected with a great number of allocated up- and down-regulated genes (16 and 12, respectively). Other pathways implicated in carbohydrate metabolism (such as pyruvate metabolism, glycolysis and gluconeogenesis, glyoxylate and dicarboxylate metabolism, pentose phosphate pathway and Citrate cycle) also presented several allocated genes.

A substantial number of genes were also mapped to KEGG pathways directly or indirectly implicated in energy metabolism (such as carbon fixation in photosynthetic organisms, nitrogen metabolism, oxidative phosphorylation and porphyrin and chlorophyll metabolism). In this context, “carbon fixation in photosynthetic organisms” was positively affected with 8 up-regulated associated genes, while “oxidative phosphorylation” was negatively affected with 6 down-regulated associated genes. Concerning lipid metabolism, the pathways “fatty acid elongation”, alpha-linolenic acid metabolism” and “fatty acid degradation” were negatively affected with uniquely down-regulated genes associated to them. Alterations in purine metabolism and in different amino acid metabolic pathways were also prominent in the AtUCP1 OE lines. In this respect, “cysteine and methionine metabolism” was the most altered, while “valine, leucine and isoleucine degradation” and “lysine degradation” were negatively affected.

Overall, these results indicate that the most important changes in gene expression detected in the AtUCP1 OE lines were mainly associated with pathways involved in carbohydrate metabolism, cell energy supply and amino acid metabolism. Collectively, these data support the proposed role of AtUCP1 in the control of carbon and nitrogen partitioning [33].

As a complementary approach, by using the pathways retrieved from the Gramene database, we identified the pathways considered to be differentially expressed between OE lines and WT. Based on this analysis, the pathways significantly up-regulated in the AtUCP1 OE lines were: Calvin-Benson-Bassham cycle (Z-score 2.1), chlorophyllide a biosynthesis I (Z-score 3.63) and tetrapyrrole biosynthesis I (Z-score 2.21). On the other hand, the pathways significantly down-regulated were: vitamin E biosynthesis (Z-score 2.5), 4-hydroxybenzoate biosynthesis V (Z-score -2) and tyrosine degradation I (Z-score -3.2).

### RT-qPCR validation of differential expression

**Fig 5:**
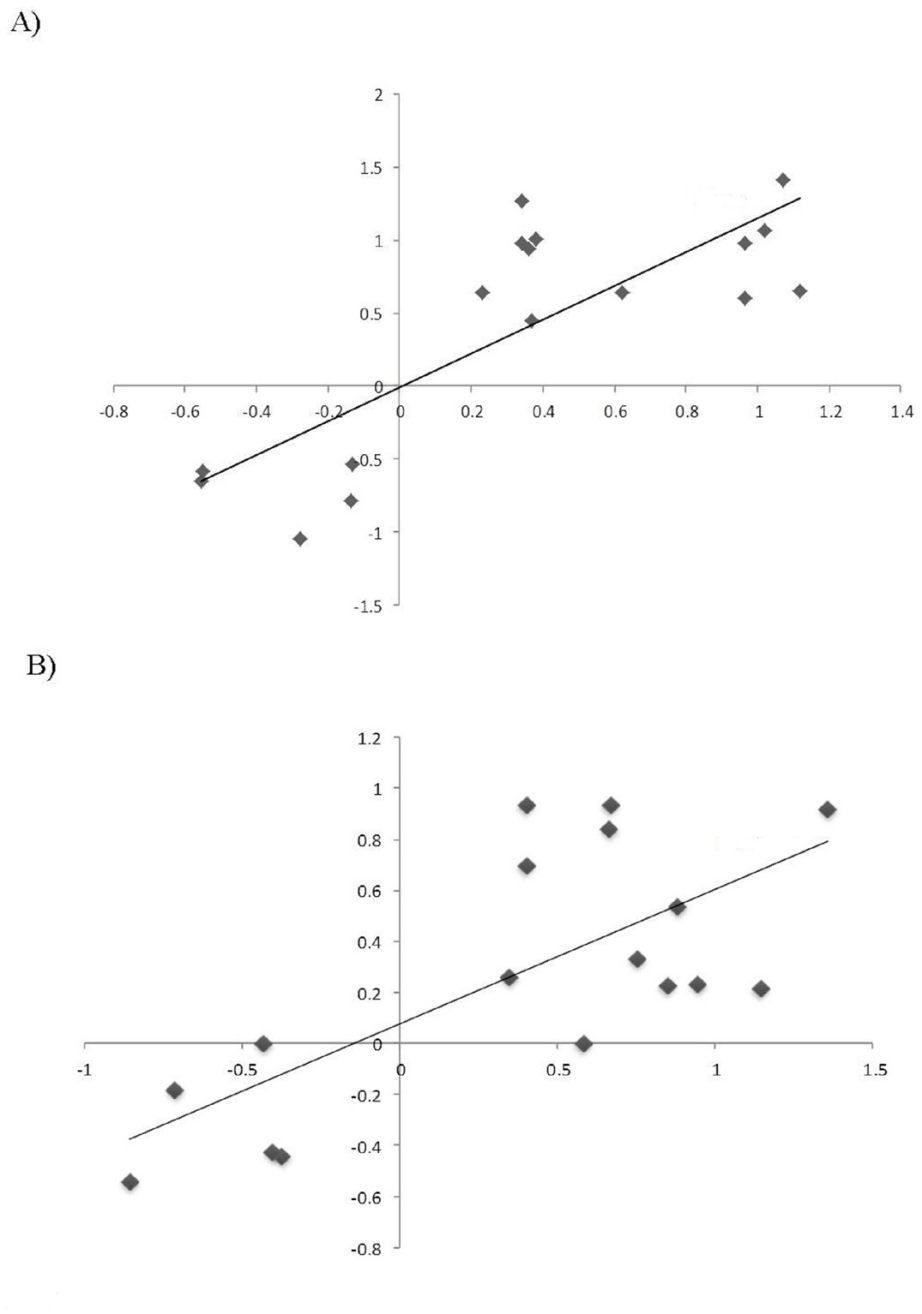
Correlation between RNA-Seq and RT-qPCR data. Twelve up-regulated and five down-regulated genes were selected for validation and a Pearson's correlation was calculated base on the expression values generated for each AtUCP1 OE line (P07 and P32).

The accuracy of the expression profiles derived from the RNA-Seq analysis was further verified using RT-qPCR. For that, 17 DEGs (12 up-regulated and 5 down-regulated; S1 Table) were chosen based on their involvement in the aforementioned altered pathways, or randomly selected among the most up and down-regulated genes. Overall, a positive correlation between the expression profiles determined in both assays was observed for each transgenic line investigated (Fig 5) (Pearson coefficient of 0.802 for P07 and 0.7212 for P32), and both correlations were significant (p<0.05).

**Fig 6:**
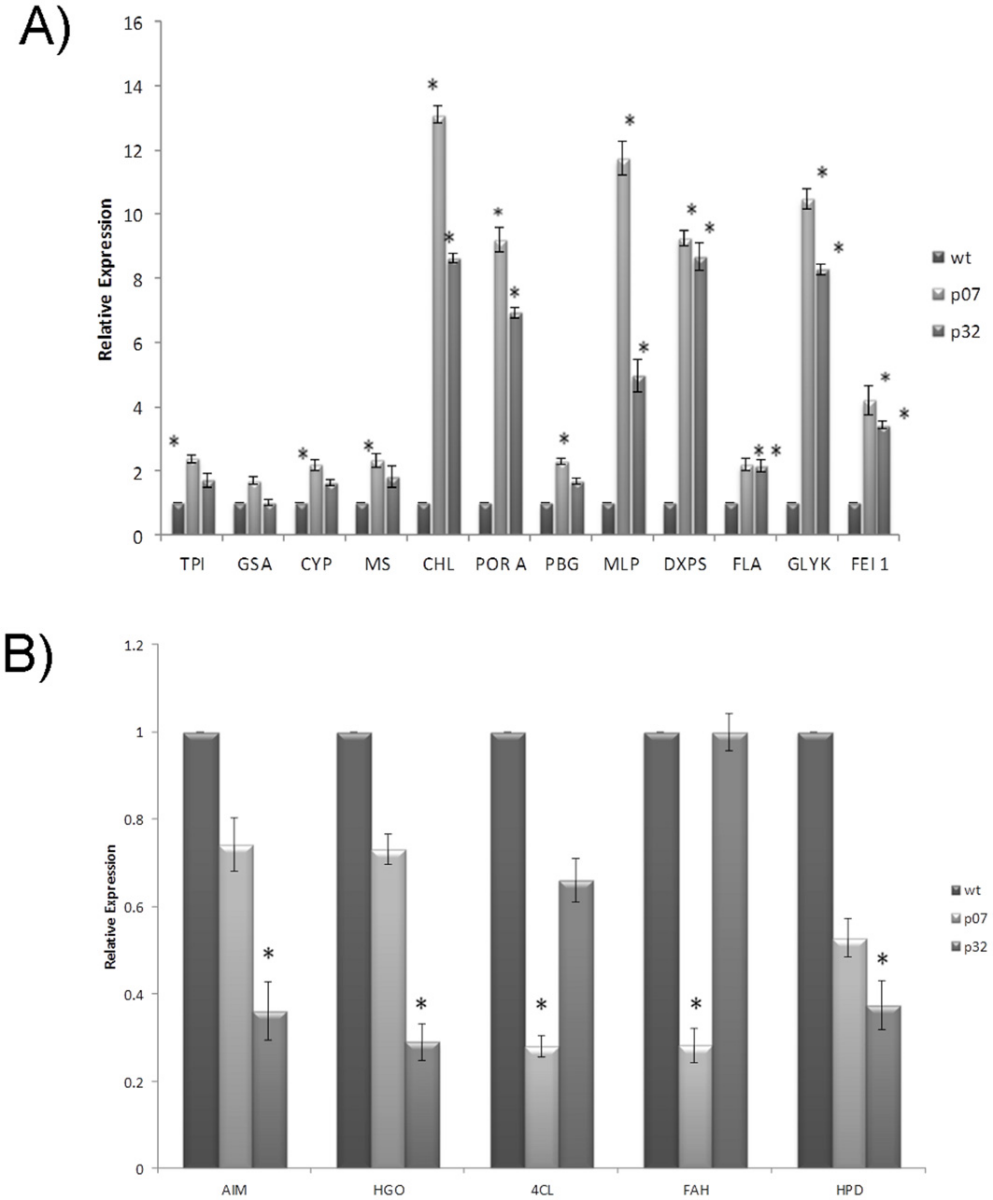
RT-qPCR validation of a set of differentially expressed genes. (A) Validation of the selected up-regulated genes. (B) Validation of the selected down-regulated genes. Assays were performed using the same RNA samples used for RNA-sequencing of both OE lines (P07 and P32) and WT expression was set to 1. Bars indicate standard errors of triplicate reactions (* means p<0.05).

According to the relative expression levels determined by RT-qPCR, all the genes considered up-regulated by the RNA-Seq analysis also showed induced expression levels in both OE lines as compared to the WT (Fig 6A). The only exception was the *GSA* gene whose relative expression was not altered. However, for three of them (*CYP*, *MS* and *PBGD*), a statistically significant up-regulation was only detected in the P07 OE line.

Concerning the validation of the selected down-regulated genes, the RT-qPCR results revealed that three of the validated genes (*AIM*, *HGO* and *HPPD*) showed low relative expression in both OE lines as compared to WT control, but were significantly down-regulated only in the P32 OE line (Fig 6B). On the contrary, the *4CL* and *FAH* genes were significantly down-regulated only in P07. Despite the observed divergences, the RT-qPCR results confirmed the down-regulation of these 5 genes in at least one of the OE lines investigated.

### Metabolite profiling

In an attempt to correlate the transcriptional alterations induced by AtUCP1 overexpression with possible metabolic changes, an untargeted metabolite profiling by GC-MS was carried out using entire seedlings of WT and AtUCP1 OE lines. By using this metabolomic approach, we were able to measure a total of 125 metabolites (S6 Table). Despite the observed differences in the average intensity of several metabolites when comparing WT and OE lines, a Volcano plot analysis revealed statistically significant changes only for Cinnamic acid 4-hydroxy-, trans- and serine, which were significantly less and more abundant in the transgenic OE lines, respectively. The observed low metabolite abundance variation is consistent with the study of Sweetlove et al. [27] showing few significant changes in metabolite levels in the leaves of the *atucp1* insertion mutant compared to WT. The increased abundance of serine in the AtUCP1 OE lines is indicative of an exacerbated photorespiration in the transgenic plants since this amino acid is an important intermediate of the photorespiratory pathway. Remarkably, a decrease in serine levels (29%) compared to WT was reported for the *atucp1* mutant that shows restricted photorespiration [27].

## Discussion

In this study we determined the global expression changes associated with the overexpression of a mitochondrial uncoupling protein (AtUCP1) in tobacco. For that, developing seedlings of two independent OE lines showing high abiotic stress tolerance and a fast growth rate during initial development were employed [9]. Our results demonstrate that AtUCP1 overexpression promotes substantial alterations in the transcriptome of the OE lines resulting in a total of 816 in common DEGs when compared to WT nontransformed plants. As a general response to UCP1 overexpression, noticeable changes in the expression of genes involved in carbon and energy metabolism were observed, indicating that an important transcriptome reprogramming is necessary to maintain energy homeostasis in the transgenic plants. Interestingly, the transcriptomic patterns identified here resemble, at least in part, those triggered by different mitochondrial dysfunctions [34,35].

Several genes encoding chloroplast-localized proteins were up-regulated in the OE lines, indicating that AtUCP1 overexpression has an important impact outside the mitochondrion as already observed for the alternative oxidase [4,36]. AtUCP1 overexpression enhanced, for example, the expression of genes encoding important subunits of the reaction centers of photosystem I and II (PSI and PSII) and also of the cytochrome *b6f* complex. Moreover, genes encoding oxygen-evolving enhancer proteins and the light-harvesting chlorophyll a/b-binding proteins are amongst the most up-regulated genes in both AtUCP1 OE lines. The detection of transcriptional changes in a significant number of photosynthesis-related genes (Fig 3A) is in line with the proposed role of UCP in maintaining rates of photosynthesis. In this respect, previous studies have demonstrated that UCP activity affects photosynthetic efficiency [13,27,28], and increase photosynthetic capacity when overexpressed in transgenic plants [8,9]. Consistently, a connection between mitochondrial function and the transcriptional regulation of nuclear genes encoding photosynthetic proteins was already evidenced in Arabidopsis and *Chlamydomonas reinhardtii* [35,37,38].

The aforementioned results strongly indicate that AtUCP1 overexpression triggers a retrograde signaling that affect photosynthetic gene expression. Particularly interesting in this context was the up-regulation of pathways associated with tetrapyrrole and chlorophyllide a biosynthesis in the AtUCP1 OE lines. Although the requirement for de novo chlorophyll biosynthesis could be associated with the elevated photosynthetic rates of the AtUCP1 OE lines [9], intermediates of such pathways (Mg-protoporphyrin IX for example) as well as changes in the flux through the tetrapyrrole pathway have been shown to play an important role in plastid-to-nucleus retrograde signaling [39,40]. Notably, porphyrins have been also postulated to participate in interorganelle signaling [39].

Earlier data reported that the photosynthetic performance of an *atucp1* insertion mutant was impaired due to the inhibition of the photorespiratory flux and limited regeneration of ribulose-1.5-bisphosphate (RuBP) [27]. In fact, many published data support the view that impaired photorespiration can restrict photosynthesis under situations that promote high oxygenation of the Rubisco enzyme, especially under stressful conditions [41]. Moreover, evidence from UCP-silenced tomato plants suggests that UCP activity affects Rubisco carboxylation and RuBP regeneration rates [28]. In this respect, the most strongly up-regulated gene in our analysis was *GLYK* (S2 Table), which encodes a key regulator of photorespiration. D-glycerate 3-kinase (GLYK) catalyzes the concluding reaction of the photorespiratory cycle and is crucial for the regeneration of RuBP [42]. It seems plausible that the detected up-regulation of *GLYK* probably represents a way to sustain the elevated photosynthetic rates displayed by the AtUCP1 OE lines [9]. Consistent with this, the Calvin-Benson cycle figured as up-regulated in the AtUCP1 OE lines indicating that RuPB does not limit cycle activity.

Another interesting finding concerning the photorespiratory pathway was the detected up-regulation of the gene encoding the mitochondrial malate dehydrogenase (mMDH). This enzyme, classically known for its role in the tricarboxylic acid (TCA) cycle, also operates during the conversion of glycine to serine by reducing oxaloacetate to malate and regenerating NAD^+^ for glycine decarboxylase [43]. In this context, Sweetlove et al. [27] showed that the photorespiratory flux of glycine to serine was substantially decreased in the *atucp1* mutant. In contrast, our metabolic profiling revealed a higher abundance of serine in the AtUCP1 OE lines. Interestingly, serine has been shown to act as a metabolic signal for the transcriptional regulation of genes involved in photorespiration [44]. It is therefore possible that the observed mMDH up-regulation in the OE lines would avoid excess NADH accumulation and sustain NAD^+^ regeneration. Of note, an enrichment of terms associated with the pentose phosphate pathway was observed (Fig 3B) and probably reflects the need of reductant availability to maintain cellular redox homeostasis in the OE lines.

UCP overexpression has been previously found to increase TCA cycle flux with no apparent alterations in the maximal catalytic activity of specific TCA cycle enzymes [12]. Among the genes involved in the TCA cycle represented in our dataset, those encoding the E1 beta and alpha subunits of the mitochondrial pyruvate dehydrogenase complex, respectively, were found to be significantly up-regulated in the OE lines (S2 Table). The PHD complex catalyzes the conversion of pyruvate into acetyl-CoA that is subsequently consumed in the TCA cycle. We also notice, as aforementioned, the up-regulation of mMDH, a classical TCA cycle enzyme that catalyzes the conversion of malate into oxaloacetate. Additionally, AtUCP1 overexpression negatively regulated the expression of a gene encoding the iron sulfur subunit 2 of the mitochondrial succinate dehydrogenase. Succinate dehydrogenase (Complex II) is a multimeric enzyme that has a dual role in mitochondrial metabolism, acting both in the TCA cycle and as a member of the respiration chain. It was previously demonstrated that plants overexpressing this subunit show high rate of photosynthesis and CO_2_ assimilation but lower rate of TCA cycle [45]. Therefore, substantial transcriptional modulations of TCA cycle-related genes are observed in the AtUCP1 OE lines, which seem likely to occur in response to an altered cycle flux. Similarly, a moderate increase in the TCA cycle enzymes was part of the response induced by the ectopic expression of mammalian UCP1 in yeast [46].

UCP has been postulated to serve a signaling role through its known implication in the control of mitochondrial ROS generation and cellular redox homeostasis [47]. In this regard, the activity of mammalian UCP2 and UCP3 has been shown to be controlled by reversible glutathionylation [48], a post-translational modification that could serve as a regulatory mechanism to govern mitochondrial ROS production and signaling [47,48]. Taking into account that the expression of different components of the photosynthetic and respiratory electron transport chains, including Complex II, has been reported to be redox-regulated [49], it is tempting to propose that the observed transcriptional modulations are associated with a ROS-mediated retrograde signaling triggered by AtUCP1 activity. Nevertheless, further experiments are needed to unravel this possibility.

In this context, by further focusing on the expression of genes involved in cellular antioxidant stress response, we observed the down-regulation of different thioredoxin, ferredoxin and nucleoredoxin in our dataset. The repression of genes involved in oxidative stress could be a direct consequence of the reported ROS-attenuating function of AtUCP1 [7,11]. In fact, AtUCP1 overexpression attenuated ROS accumulation in the investigated OE lines [9,10]. Overall, these findings further strength the proposed role of UCP in redox homeostasis and signaling.

Surprisingly, despite the already described reduction in cellular ATP concentration (20–35%) provoked by the dissipating action of AtUCP1 in leaves of the P07 OE line [10], we observed no changes in ATP levels measured in intact AtUCP1 OE seedlings compared to WT. Thereafter, it seems that ATP level is not functioning as a major retrograde signal in developing seedlings as previously proposed for P07 line leaves [10]. The fact that an opposite regulation of the genes encoding the PSI and PSII core proteins (unaltered) and the light-harvesting complex II (repressed) was observed in Arabidopsis lines with elevated ATP content [50] argues in favor of this idea. Also consistent with this, none of the genes encoding the major components of the mitochondrial respiratory chain complexes figured as up-regulated in our datasets. On the contrary, the expression of a number of genes coding for components of complex I, complex II and the beta subunit of ATP synthase was significantly down-regulated in both OE lines (S2 and S5 Tables). The reason for this is unclear but Umbach et al. [36] also observed no changes in the transcript abundance of selected electron transport components in the leaves of normally grown transgenic Arabidopsis overexpressing another component of the mitochondrial alternative pathway (AOX1a). Our results are well in line with the undetected differences in the respiration (measured as CO_2_ release) of the AtUCP1 OE lines and WT plants [9], but contrast with those obtained by Barreto et al. [10] showing an up-regulation of both nuclear-encoded (NADH-DeH and NADH-IS7) and mitochondrial-encoded (NADH-IS2) components of complex I in the leaves of 12-week-old P07 plants. These discrepancies could be attributed to differences in the developmental stage and overall metabolic and physiological status of the sampled material (intact seedlings vs. leaves), and point to the existence of additional posttranscriptional and posttranslational mechanisms as proposed [36].

In conclusion, the transcriptional profiling presented here provides novel insights into the molecular responses brought about by the augmented expression of an uncoupling protein in plants. The overall results also highlight the occurrence of an important transcriptional cross-talk between chloroplast and mitochondria, which culminates in the transcriptional regulation of genes mainly involved in photosynthesis, photorespiration and carbon metabolism.

## Supporting Information

S1 Fig Network visualization of the hierarchical relationships among GO “biological process” terms. Colored nodes are significantly overrepresented GO categories (node colors represent enrichment significance as depicted in Fig 4) associated with the up-regulated gene dataset. The branches represented in Fig 4 are indicated. Due to its large size, this figure is best viewed with a zoom of 1600%. Data were analyzed using the Biological Networks Gene Ontology tool (BiNGO).
(PDF)

S1 Table Genes selected for expression validation by RT-qPCR with corresponding primers and detected RNA-Seq fold changes. (DOCX)

S2 Table List of differentially expressed genes. Fold change indicates the average of up- or down-regulation in the *AtUCP1* OE lines compared with WT.
(XLSX)

S3 Table Significantly enriched GO terms for up-regulated genes. (DOC)

S4 Table Significantly enriched GO terms for down-regulated genes. (DOC)

S5 Table KEGG metabolic pathways altered in the *AtUCP1* OE lines (only pathways with 5 or more affected genes are represented). (XLSX)

S6 Table Average intensities of the metabolites found in the WT and AtUCP1 OE lines. (XLSX)
