# Supplementary figures and images for: Transcriptome Response Signatures Associated with the Overexpression of a Mitochondrial Uncoupling Protein (AtUCP1) in Tobacco
Source: PLoS One. 2015 Jun 24;10(6):e0130744. doi: 10.1371/journal.pone.0130744 (PMC4479485; doi:10.1371/journal.pone.0130744)

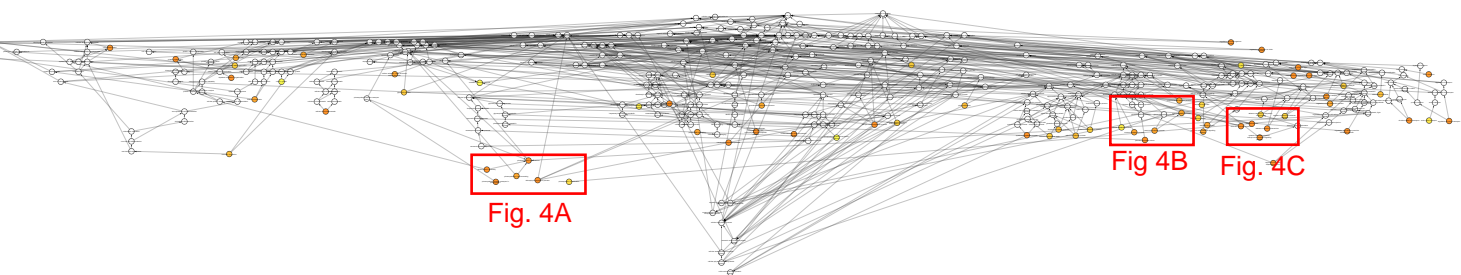

Supplement: S1 Fig — Colored nodes are significantly overrepresented GO categories (node colors represent enrichment significance as depicted in Fig 4) associated with the up-regulated gene dataset. The branches represented in Fig 4 are indicated. Due to its large size, this figure is best viewed with a zoom of 1600%. Data were analyzed using the Biological Networks Gene Ontology tool (BiNGO). (PDF) [file pone.0130744.s001.pdf]
