# Supplementary material for: Transcriptome Response Signatures Associated with the Overexpression of a Mitochondrial Uncoupling Protein (AtUCP1) in Tobacco
Source: PLoS One. 2015 Jun 24;10(6):e0130744. doi: 10.1371/journal.pone.0130744 (PMC4479485; doi:10.1371/journal.pone.0130744)
Supplement: S1 Table — (DOCX) [file pone.0130744.s002.docx]

Table S1. Genes selected for expression validation by RT-qPCR and corresponding primers and detected RNA-Seq fold changes.

| **Gene** | **Fold RNA-Seq (P07/P32)** | **Primer pair** | **Primer Sequence** |  |  |
| --- | --- | --- | --- | --- | --- |
| Triosephosphate isomerase (TPI) | 10.15/8.79 | TPI-F | 5'-ATTGACCAACATCTCAGCACTAA-3' | | |
|  |  | TIP-R | 5'-TGCGTCCTGATTTCTCCGTT-3' | | |
| Glutamate-1-semialdehyde aminotransferase (GSA) | 4.37/3.79 | GSA-F | 5'-CTGTTCGCGCTTTCAAGTCA-3' |  |  |
|  |  | GSA-R | 5'-GCCCTTCACGGAGTCAATAATTG-3' |  |  |
| Cytochrome p450 77a3-like (CYP77A3) | 18.78/13.78 | CYP-F | 5'-CAAATTCAGTGTCAATGCAGCAA-3' |  |  |
|  |  | CYP-R | 5'-GCAACTAAGCATATTTTGGACCATATT-3' |  |  |
| Methionine synthase (MS) | 2.82/2.23 | MS-F | 5'-TCACTGCTTTGAAGGGAGTTA-3' | | |
|  |  | MS-R | 5'-CACTCCTGCAAACAAGTACTTT-3' | | |
| Mg protoporphyrin IX chelatase (Chl H) | 4.46/3.77 | CHL-F | 5'-CCAAAGGCAATGCCACAATA-3' |  |  |
|  |  | CHL-R | 5'-CAAGACCATCCAAGGGCAAT-3' |  |  |
| Protochlorophyllide oxidoreductase (POR-A) | 4.03/4.61 | POR A-F | 5'-ATTAGAGCCGAGACAATGGTT-3' | | |
|  |  | POR A-R | 5'-AATCCTGAAGAGGCTCCAGTAA-3' | | |
| Porphobilinogen deaminase (PBGD) | 8.73/7.05 | PBG-F | 5'-GAGGCAACCAATCCTTTGAAAA-3' |  |  |
|  |  | PBG-R | 5'-GCACCCCAATTGCTGGATAT-3' |  |  |
| Major latex protein-like 28 (MLP28) | 26.18/2.52 | MLP-F | 5'-TTCCCAGTTAGGTGATGAGTCTCA-3' |  |  |
|  |  | MLP-R | 5'-TCCACACACATTCCTGCAATTCTG-3' |  |  |
| 1-deoxy-d-xylulose-5-phosphate (DXPS) | 5.1/4.62 | DXPS-F | 5'-ACGGATCTCCTGCTGATCAAT-3' | | |
|  |  | DXPS-R | 5'-TATGTCATAACCTCTAGAGCTTCT-3' | | |
| Fasciclin-like arabinogalactan protein 17 (FLA) | 9.45/5.66 | FLA-F | 5'-TCATAGCACTGCTCACCATTTTC-3' |  |  |
|  |  | FLA-R | 5'-TGCACGCGACCTTGTATCC-3' |  |  |
| D-glycerate 3-kinase (GLYK) | 11.65/22.69 | GLYK-F | 5'-CCACACTCTACTCAGAAGGACCTACTG-3' |  |  |
|  |  | GLYK-R | 5'-ATTTCTCCCTTCATCGATTTCAAT-3' |  |  |
| LRR receptor-like serine/threonine-protein kinase | 4.35/7.55 | FEI 1-F | 5'-CAACTCTTGAGAAAATGAAAAGCA-3' |  |  |
|  |  | FEI 1-R | 5'-GGGCGGCTTCTCTTTGAAAA-3' |  |  |
| Peroxisomal fatty acid beta-oxidation multifunctional protein aim 1-like (AIM) | -3.44/-2.38 | AIM-F | 5'-GCAGAAAGGCGCAAACCTT-3' |  |  |
|  |  | AIM-R | 5'-GACTTCAGTACCTCCCGTGCTT-3' |  |  |
| Homogentisate 1,2-dioxygenase (HGO) | -6.17/-7.12 | HGO-F | 5'-ACACGACGCTCTTCCGATCT-3' |  |  |
|  |  | HGO-R | 5'-CATTGATGGTTTGTACACGATATGC-3' |  |  |
| 4-hydroxyphenylpyruvate dioxygenase-like (HPPD) | -11.02/-2.54 | HPD-F | 5'-GCGGTTTGTGAGCTATCTGAAA-3' |  |  |
|  |  | HPD-R | 5'-TCCTCCATGGCCTCGGAAA-3' |  |  |
| 4-coumarate-- ligase-like 7-like (4CLL7) | -4.47/-5.17 | 4CL-F | 5'-GCACCTTTAGGGAAGGACTT-3' | | |
|  |  | 4CL-R | 5'-ACAATGTTTCTGTCATGCCATA-3' | | |
| Fumarylacetoacetase (FAH) | -3.89/-2.70 | FAH-F | 5'-TTAGAAGATGGAGATGAAGTAACTTT-3' |  |  |
|  |  | FAH-R | 5'-ACAATGTTAGTAATTCATGCCATTGT-3' |  |  |
| Elongation factor (EF) | Control | NtEF-F | 5'- TCTGATCCATTGGATGCTGG-3' |  |  |
|  |  | NtEF-R | 5'-TTCAAACCCTTCCTCTTGCG-3' |  |  |
