# Supplementary material for: Transcriptome Response Signatures Associated with the Overexpression of a Mitochondrial Uncoupling Protein (AtUCP1) in Tobacco
Source: PLoS One. 2015 Jun 24;10(6):e0130744. doi: 10.1371/journal.pone.0130744 (PMC4479485; doi:10.1371/journal.pone.0130744)
Supplement: S3 Table — (DOC) [file pone.0130744.s004.doc]

Table S3 - Significantly enriched GO terms for up-regulated genes.

| **BIOLOGICAL PROCESS** |  |  |  |
| --- | --- | --- | --- |
| GO-ID | FDR | Description | Annotated transcripts |
| 6098 | 2.98E-10 | pentose-phosphate shunt | 26 |
| 6364 | 2.98E-10 | rRNA processing | 24 |
| 19344 | 5.66E-10 | cysteine biosynthetic process | 22 |
| 19684 | 6.40E-10 | photosynthesis, light reaction | 14 |
| 10207 | 5.57E-08 | photosystem II assembly | 17 |
| 10114 | 5.57E-08 | response to red light | 16 |
| 9637 | 5.57E-08 | response to blue light | 16 |
| 10218 | 1.56E-07 | response to far red light | 16 |
| 15995 | 2.85E-07 | chlorophyll biosynthetic process | 16 |
| 9409 | 7.43E-07 | response to cold | 26 |
| 9773 | 1.40E-06 | photosynthetic electron transport in photosystem I | 10 |
| 35304 | 2.17E-06 | regulation of protein dephosphorylation | 13 |
| 9657 | 6.40E-06 | plastid organization | 8 |
| 9697 | 1.65E-05 | salicylic acid biosynthetic process | 12 |
| 42742 | 2.60E-05 | defense response to bacterium | 20 |
| 70838 | 3.45E-05 | divalent metal ion transport | 9 |
| 19288 | 4.50E-05 | isopentenyl diphosphate biosynthetic process | 17 |
| 10310 | 9.25E-05 | regulation of hydrogen peroxide metabolic process | 10 |
| 30003 | 1.04E-04 | cellular cation homeostasis | 9 |
| 16117 | 1.08E-04 | carotenoid biosynthetic process | 12 |
| 10027 | 1.88E-04 | thylakoid membrane organization | 15 |
| 10155 | 5.78E-04 | regulation of proton transport | 9 |
| 9595 | 8.23E-04 | detection of biotic stimulus | 7 |
| 9862 | 1.34E-03 | systemic acquired resistance | 10 |
| 43900 | 1.34E-03 | regulation of multi-organism process | 7 |
| 2831 | 1.34E-03 | regulation of response to biotic stimulus | 7 |
| 30154 | 1.56E-03 | cell differentiation | 9 |
| 6636 | 2.42E-03 | unsaturated fatty acid biosynthetic process | 8 |
| 43085 | 2.92E-03 | positive regulation of catalytic activity | 9 |
| 6833 | 3.00E-03 | water transport | 11 |
| 19252 | 9.19E-03 | starch biosynthetic process | 11 |
| 1900160 | 9.19E-03 | plastid DNA packaging | 2 |
| 19424 | 9.19E-03 | sulfide oxidation, using siroheme sulfite reductase | 2 |
| 9744 | 9.58E-03 | response to sucrose stimulus | 11 |
| 5985 | 9.60E-03 | sucrose metabolic process | 15 |
| 9867 | 1.05E-02 | jasmonic acid mediated signaling pathway | 9 |
| 9965 | 1.05E-02 | leaf morphogenesis | 11 |
| 7389 | 1.05E-02 | pattern specification process | 5 |
| 8361 | 1.05E-02 | regulation of cell size | 5 |
| 9765 | 1.05E-02 | photosynthesis, light harvesting | 4 |
| 19216 | 1.05E-02 | regulation of lipid metabolic process | 4 |
| 19761 | 1.17E-02 | glucosinolate biosynthetic process | 9 |
| 272 | 1.18E-02 | polysaccharide catabolic process | 5 |
| 5982 | 1.19E-02 | starch metabolic process | 15 |
| 9853 | 2.03E-02 | photorespiration | 9 |
| 9902 | 2.05E-02 | chloroplast relocation | 8 |
| 9099 | 2.05E-02 | valine biosynthetic process | 5 |
| 9098 | 2.05E-02 | leucine biosynthetic process | 5 |
| 48767 | 2.10E-02 | root hair elongation | 10 |
| 6096 | 2.61E-02 | glycolysis | 15 |
| 19676 | 2.70E-02 | ammonia assimilation cycle | 2 |
| 15976 | 2.84E-02 | carbon utilization | 7 |
| 9664 | 2.90E-02 | plant-type cell wall organization | 7 |
| 9073 | 2.90E-02 | aromatic amino acid family biosynthetic process | 5 |
| 9097 | 2.90E-02 | isoleucine biosynthetic process | 5 |
| 43086 | 2.90E-02 | negative regulation of catalytic activity | 5 |
| 10143 | 2.90E-02 | cutin biosynthetic process | 3 |
| 31348 | 3.00E-02 | negative regulation of defense response | 8 |
| 9814 | 3.11E-02 | defense response, incompatible interaction | 4 |
| 9749 | 3.13E-02 | response to glucose stimulus | 6 |
| 9750 | 3.31E-02 | response to fructose stimulus | 8 |
| 42549 | 3.52E-02 | photosystem II stabilization | 2 |
| 9926 | 3.87E-02 | auxin polar transport | 6 |
| 38 | 4.14E-02 | very long-chain fatty acid metabolic process | 4 |
| 10037 | 4.14E-02 | response to carbon dioxide | 2 |
| 23 | 4.23E-02 | maltose metabolic process | 8 |
| 55114 | 4.76E-02 | oxidation-reduction process | 28 |
| 9932 | 4.85E-02 | cell tip growth | 6 |
| 6655 | 4.88E-02 | phosphatidylglycerol biosynthetic process | 5 |
| **MOLECULAR FUNCTION** | |  |  |
| **GO-ID** | **FDR** | **Description** | Annotated transcripts |
| 16720 | 0.018 | delta12-fatty acid dehydrogenase activity | 3 |
| CELLULAR COMPONENT | |  |  |
| GO-ID | FDR | Description | Annotated transcripts |
| 9941 | 7.70E-17 | chloroplast envelope | 50 |
| 48046 | 1.87E-16 | apoplast | 37 |
| 9535 | 3.18E-15 | chloroplast thylakoid membrane | 32 |
| 9570 | 3.11E-10 | chloroplast stroma | 42 |
| 9543 | 5.78E-07 | chloroplast thylakoid lumen | 10 |
| 10287 | 2.93E-06 | plastoglobule | 11 |
| 9654 | 9.89E-06 | oxygen evolving complex | 6 |
| 10319 | 5.04E-04 | stromule | 8 |
| 19898 | 5.36E-04 | extrinsic to membrane | 5 |
| 30095 | 6.75E-04 | chloroplast photosystem II | 4 |
| 9505 | 8.18E-03 | plant-type cell wall | 12 |
| 9538 | 4.13E-02 | photosystem I reaction center | 2 |
