# Supplementary material for: Transcriptome Response Signatures Associated with the Overexpression of a Mitochondrial Uncoupling Protein (AtUCP1) in Tobacco
Source: PLoS One. 2015 Jun 24;10(6):e0130744. doi: 10.1371/journal.pone.0130744 (PMC4479485; doi:10.1371/journal.pone.0130744)
Supplement: S4 Table — (DOC) [file pone.0130744.s005.doc]

| **BIOLOGICAL PROCESS** | |  |  |
| --- | --- | --- | --- |
| **GO-ID** | **FDR** | **Description** | **Annotated transcripts** |
| 15996 | 2.55E-04 | chlorophyll catabolic process | 12 |
| 46686 | 3.33E-04 | response to cadmium ion | 53 |
| 6457 | 4.87E-03 | protein folding | 26 |
| 9407 | 3.43E-02 | toxin catabolic process | 14 |
| 6979 | 3.80E-02 | response to oxidative stress | 18 |
| 6635 | 3.80E-02 | fatty acid beta-oxidation | 17 |
| 18874 | 3.80E-02 | benzoate metabolic process | 8 |
| 19458 | 3.80E-02 | methionine catabolic process via 2-oxobutanoate | 2 |
| 42313 | 3.80E-02 | protein kinase C deactivation | 2 |
| 6972 | 4.26E-02 | hyperosmotic response | 13 |
| 9414 | 4.62E-02 | response to water deprivation | 19 |
| 6623 | 4.62E-02 | protein targeting to vacuole | 14 |
| 6643 | 4.62E-02 | membrane lipid metabolic process | 3 |
| **CELLULAR COMPONENT** | |  |  |
| **GO-ID** | **FDR** | **Description** | **Annotated transcripts** |
| 5829 | 4.58E-04 | cytosol | 100 |
| 9506 | 9.58E-03 | plasmodesma | 46 |
| 44428 | 2.31E-02 | nuclear part | 3 |
| 5886 | 2.87E-02 | plasma membrane | 110 |
| 30136 | 2.87E-02 | clathrin-coated vesicle | 3 |

Table S4 - Significantly enriched GO terms for down-regulated genes.
